# Acquisition of Quasi-Monochromatic Dual-Energy in a Microfocus X-ray Generator and Development of Applied Technology

**DOI:** 10.3390/diagnostics9010027

**Published:** 2019-03-04

**Authors:** Hiroaki Hasegawa, Masanori Sato

**Affiliations:** 1 Department of Radiology, The University of Tokyo Hospital, Bunkyo-ku, Tokyo 113-8655, Japan; 2 Department of Radiological Sciences, Faculty of Health Sciences, Komazawa University, Setagaya-ku, Tokyo 154-8525, Japan; masasato@komazawa-u.ac.jp

**Keywords:** microfocus X-ray generator, DEXA, K-absorption edge, dual-energy X-ray, bone mineral density

## Abstract

In regenerative medicine, evaluation of bone mineral density using a microfocus X-ray generator could eventually be used to determine the degree of bone tissue regeneration. To evaluate bone mineral density against regenerated bone material, two low-energy X-rays are necessary. Herein, the acquisition of quasi-monochromatic, dual-energy soft X-ray and the subsequent medical application were examined using the K-absorption edges of two types of metal filters (i.e., zirconium and tin) in a microfocus X-ray generator. Investigation of the optimal tube voltage and filter thickness to form a quasi-monochromatic energy spectrum with a single filter revealed that a filter thickness of 0.3 mm results in an optimal monochromatization state. When a dual filter was used, the required filter thickness was 0.3 mm for tin and 0.2 mm for zirconium at a tube voltage of 35 kV. For the medical application, we measured quasi-monochromatic, dual-energy X-rays to evaluate the measurement accuracy of bone mineral density. Using aluminum as a simulated bone sample, a relative error of ≤5% was consistent within the aluminum thickness range of 1–3 mm. These data suggest that a bone mineral density indicator of recycled bone material can be easily obtained with the quasi-monochromatic X-ray technique using a microfocus X-ray generator.

## 1. Introduction

Development of microfocus X-ray computed tomography (CT) devices has been ongoing since the 1980s and these devices have been widely used for the non-destructive examination of industrial materials. In clinical medicine, these devices are used as X-ray devices to evaluate biomaterials (trabecular bone and implant contact) owing to their low invasiveness and high accuracy [1,2,3]. In a microfocus X-ray CT device, the focus size at which X-rays are generated is 1/100 or less than that used for a medical X-ray. Moreover, regarding having a transmission-type target to obtain high spatial resolution, its required performance significantly differs from a medical CT device [4].

In addition to the morphological evaluation of biomaterials involving optimal use of high-resolution imaging [5,6,7,8,9,10], in medical measurements using a microfocus X-ray CT device, bone mineral density (bone thickness) of regenerated bone tissues can be evaluated in regenerative medicine [11]. Because most regenerated bone tissues show low absorption of X-rays, use of low-energy X-rays (soft X-rays) is considered effective [12]. Therefore, evaluation of bone mineral density using low-energy X-ray with a microfocus X-ray CT device poses a novel measurement system for evaluating the degree of bone tissue regeneration.

Dual-energy X-ray absorptiometry (DEXA) [13,14,15,16] is used to evaluate bone mineral density and diagnose osteoporosis. DEXA is a method [17,18] that employs a single metal filter (K-absorption edge filter) to obtain two types of X-ray energy and facilitates measurement of the tube voltage at a high speed [19]. However, because effective energy varies before and after the transmission of a subject, these methods may deteriorate the accuracy of quantification [20]. To solve these problems related to effective energy, a novel technique involving quasi-monochromatization of low-energy X-rays is necessary. Synchrotron radiation is a representative monochromatic X-ray source [21,22], but in consideration of general versatility, a simple monochromatization technology independent of large facilities is required.

Therefore, we focused on quasi-monochromatic, dual-energy X-rays using two types of metal filters as a more convenient method to promote monochromatic X-ray technology at a technically feasible level in the field of tissue engineering. Considering the low cost of metal filters, they can be easily mounted on a microfocus X-ray generator, making them highly versatile. The aim of this study was to obtain quasi-monochromatic, dual-energy X-rays with two types of metal filters against low-energy X-rays obtained from a microfocus X-ray generator. We aimed to determine the best conditions for obtaining quasi-monochromatic, dual-energy X-rays by obtaining optimal quasi-monochromatic, single-energy X-rays. In addition, we aimed to evaluate the measurement accuracy of mineral density of simulated bone tissue using DEXA with quasi-monochromatic dual-energy X-rays. 

## 2. Materials and Methods

### 2.1. X-ray Spectrum Measurement

**Figure 1:**
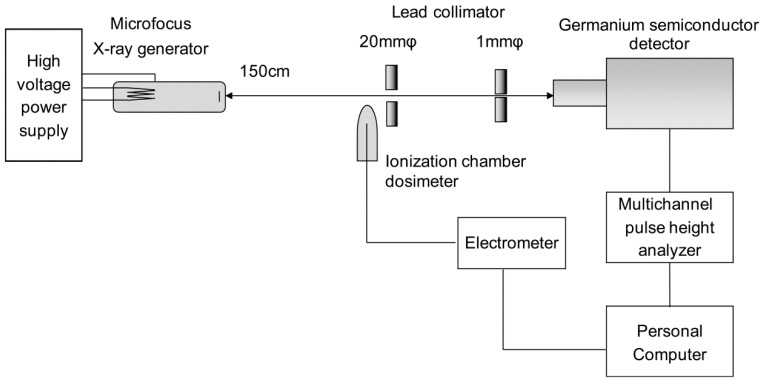
Geometric arrangement in X-ray spectrum measurement and measurement device block diagram.

The geometry of spectral measurement using a liquid nitrogen-cooled germanium semiconductor detector and the block diagram of the measuring instrument are shown in Figure 1. A microfocus X-ray source (Hamamatsu Photonics K.K., Shizuoka, Japan) was used for the X-ray tube. The target material was tungsten, and a 0.2-μm beryllium window was attached. The focus–detector distance was set at 30 cm, and two lead pinhole collimators of 1-mm diameter were placed on the X-ray central axis to form the X-ray flux incident on the detector. The K-absorption edge filter was placed on the position closest to the X-ray tube side. 

Further, the semiconductor detector used was GR1018 (Mirion Technologies, Inc., Meriden, CT, USA), whereas the multichannel pulse height analyzer used was DSA-1000 (Mirion Technologies, Inc., Meriden, CT, USA). Using the spectral analysis software Genie 2000 V3.1 provided by the same company, the currently used device facilitates operation at various settings, such as high voltage and discrimination levels on a personal computer. To maintain constant intensity of the X-ray flux radiated from the X-ray generator, the ionization chamber dosimeter DC 300 (Scanditronix Wellhöfer, Schwarzenbruck, Germany) was used with an ionization volume of 3 cm^3^ and the RAMTEC 1500 (Toyo Medic Co., Ltd, Shinjuku, Tokyo, Japan) electrometer; the tube current was adjusted to 1 pA/s. Dead time was set at 3% as a guide such that pile up was avoided due to an excessive number of photons entering the detector. To address the influence of electrical noise, a lower-level discriminator was set to 68 channels (3.0 keV), corresponding to 1.7% of all channels. The measurement was terminated when the maximum X-ray intensity reached 3000 counts. The obtained X-ray spectrum was subjected to response correction for the detector by stripping [23].

### 2.2. K-Absorption Edge Filter

The ideal and practical metal filter is inexpensive and not corrosive, irritating, or toxic. Considering K-absorption edge energy, metals known to have a K-absorption edge with a low X-ray energy of approximately 30 keV or less include zirconium (Z = 40), molybdenum (Z = 42), silver (Z = 47), indium (Z = 49), and tin (Z = 50). In the present study, we investigated zirconium and tin with the highest difference in terms of K-absorption edge energy, considering the acquisition of dual-energy X-ray spectra. The K-absorption edge energy of zirconium and tin is 17.9976 and 29.2001 keV, respectively.

### 2.3. Conditions to Generate Quasi-Monochromatic Single-Energy X-rays (Tube Voltage and Filter Thickness Dependence)

Using the spectrum calculation program provided in IPEM Report 78 [24,25,26], we examined the optimal filter thickness for a dual filter of zirconium and tin, and the results revealed that the optimal filter thickness is 0.2–0.3 mm.

To obtain the optimal condition for acquiring the X-ray spectrum, we first investigated the tube voltage that can provide quasi-monochromatic single-energy X-rays with a single filter of 0.3-mm thickness. In consideration of the K-absorption edge energy, the X-ray spectrum was measured at an interval of 10 kV in the range of 20–50 kV for the zirconium filter and 30–50 kV for the tin filter. The obtained X-ray spectra were evaluated using the following: full width at half maximum (FWHM) of the energy peak formed near the K-absorption edge energy and X-ray intensity ratio calculated according to Equation (1) (*I_K_*/*I*). *I_K_*/*I* was defined as the X-ray intensity ratio of the energy region lower than the K-absorption edge energy (i.e., energy peak of interest) and the total energy region. Based on these values, the optimal tube voltage for obtaining a quasi-monochromatic single-energy X-ray in each filter was determined with the following equation:

(1) $$\frac{I_{K}}{I}=\frac{\int_{0}^{E_{K}}I\left(E\right)dE}{\int_{0}^{E_{max}}I\left(E\right)dE}$$

where *E_max_* is the maximum energy of the X-ray, *E_K_* is the K-absorption edge energy (*E_K,Zr_* for zirconium and *E_K,Sn_* for tin), and *I*(*E*) represents the X-ray intensity at energy *E*.

Next, using the tube voltage determined from the above equation, the X-ray spectrum was measured with a filter thickness set at 0.1, 0.3, and 0.6 mm. Because filter thickness substantially affects the measurement time of the X-ray spectrum, the optimal filter thickness was determined in consideration of the FWHM and measurement time of the energy peak *I*(*E_K_*).

### 2.4. Conditions to Achieve Quasi-Monochromatic Dual-Energy

Regarding tube voltage, the intermediate value of the optimal tube voltage for obtaining quasi-monochromatic, single-energy X-rays in each filter was considered the tube voltage. It was assumed that the filter thickness would be 0.2–0.3 mm based on the spectrum calculation program. Thus, the spectral measurement was performed with a zirconium filter thickness of 0.1, 0.2, and 0.3 mm, and tin filter thickness of 0.2 and 0.3 mm; these were shown to have a marked attenuation effect. Upon setting the FWHM value of the energy peak (*I*(*E_K,Zr_*) or *I*(*E_K,Sn_*)) formed in the vicinity of the K-absorption edge of each filter and the X-ray intensity ratio of the zirconium filter to the tin filter as *I*(*E_K,Zr_*)/*I*(*E_K,Sn_*), the filter thickness condition for obtaining the quasi-monochromatic single-energy X-ray was used when this value was the largest. 

### 2.5. Evaluation of the Measurement Accuracy of Mineral Density of Simulated Bone Tissue Using DEXA

To quantitatively evaluate bone mineral density using quasi-monochromatic, dual-energy X-rays obtained from the zirconium and tin filters, we conducted a simulation experiment, simulating soft tissue with polymethyl methacrylate resin and bone with aluminum (2.70 g/cm^3^). The measurement and geometric conditions of the X-ray spectrum were the same as those of Section 2.1, and the sample was placed in the position closest to the X-ray tube side. The tube voltage and filter thickness for obtaining quasi-monochromatic, dual-energy X-ray were the conditions established in Section 2.4. Bone mineral density was calculated using Equation (2) in accordance with the measurement principle of DEXA [14,15,16].

(2) $$t_{b}\cdot \rho_{b}=\frac{ln\left(\frac{I_{0L}}{I_{L}}\right)\cdot \mu_{sH}-ln\left(\frac{I_{0H}}{I_{H}}\right)\cdot \mu_{sL}}{\mu_{sH}\cdot \mu_{bL}-\mu_{sL}\cdot \mu_{bH}}$$

where bone mineral density is defined as *t_b_*·*ρ_b_* (g/cm^2^) and *μ* is the mass attenuation coefficient (cm^2^/g). The intensity of X-rays transmitted through only the soft tissue of thickness *t_S_* (cm) at density *ρ_S_* (g/cm^3^) without penetrating bone was defined as *I*_0_, and the intensity of X-rays transmitted through bone (including soft tissue) of thickness *t_b_* at density *ρ_b_* was set as *I*. The subscripts *L* and *H* indicate the K-absorption edge energy on the low and high energy sides, respectively.

In the two quasi-monochromatic X-ray spectra obtained, the photon count of the channel corresponding to the K-absorption edge energy of zirconium and tin was used. In the DEXA method, it is necessary to change the X-ray intensity when the sample is measured. Therefore, measurements were performed until the maximum X-ray intensity was obtained (3000 counts) when there was no sample, and the X-ray spectrum when the sample was inserted at the required time was then measured. The thickness of the acrylic layer was maintained at 5 mm, but the thickness of the aluminum layer varied (0.1, 0.5, 1.0, 2.0, and 3.0 mm). The measurement was performed twice for each aluminum thickness, and aluminum density was obtained from the mean value of the photon count. Measurement accuracy was evaluated by the relative error between the value obtained from the measured X-ray spectrum and nominal value. The mass attenuation coefficient was calculated using the interpolation of values cited from the U.S. National Institute of Standards and Technology website [27].

## 3. Results

### 3.1. Tube Voltage Dependence of Quasi-Monochromatic Single-Energy X-Ray

**Figure 2:**
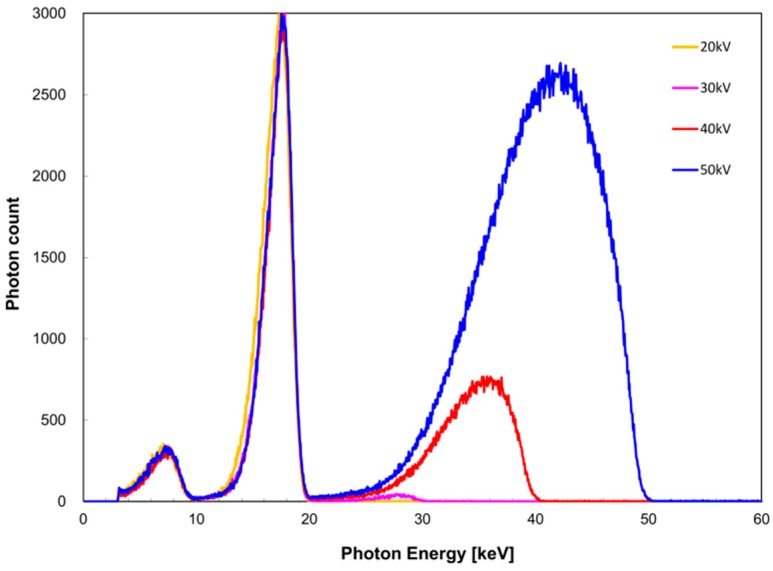
Tube voltage dependence of X-ray spectrum when the zirconium filter was used.

**Figure 3:**
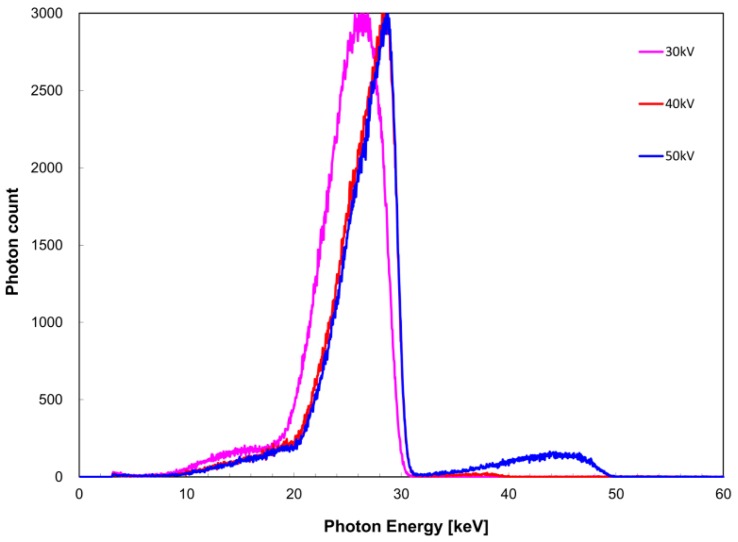
Tube voltage dependence of X-ray spectrum when the tin filter was used.

The measured X-ray spectra for the tube voltage are shown in Figure 2 for the zirconium filter and Figure 3 for the tin filter. FWHM with respect to *I*(*E_K,Zr_*) was 2.8 and 2.5 keV at a tube voltage of 20 and ≥30 kV, whereas that with respect to *I*(*E_K,Sn_*) was 6.3, 5.2, and 4.8 keV at a tube voltage of 30, 40, and 50 kV, respectively. For the zirconium filter, *I_K_*/*I* was 1.0, 0.98, 0.61, and 0.22 at a tube voltage of 20, 30, 40, and 50 kV, respectively. For the tin filter, *I_K_*/*I* was 1.0, 0.99, and 0.92 at a tube voltage of 30, 40, and 50 kV, respectively.

### 3.2. Filter Thickness Dependence of Quasi-Monochromatic Single-Energy X-ray

**Figure 4:**
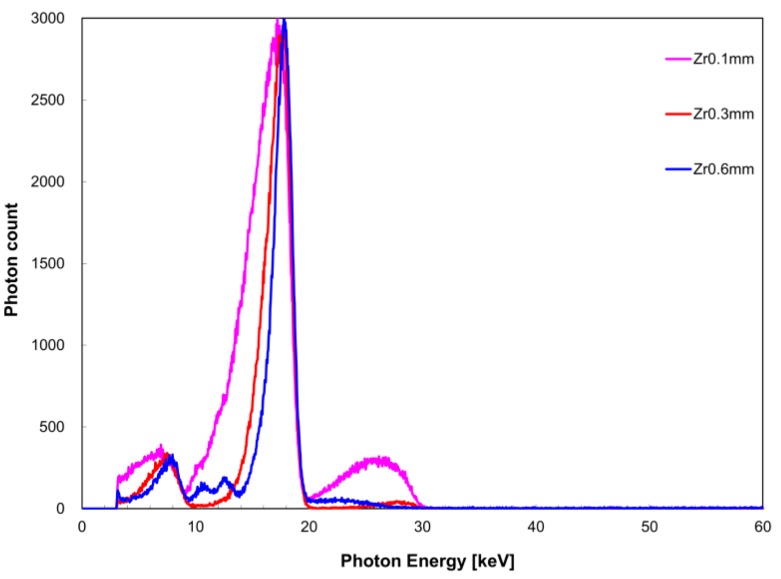
Filter thickness dependence of X-ray spectrum when the zirconium filter was used.

**Figure 5:**
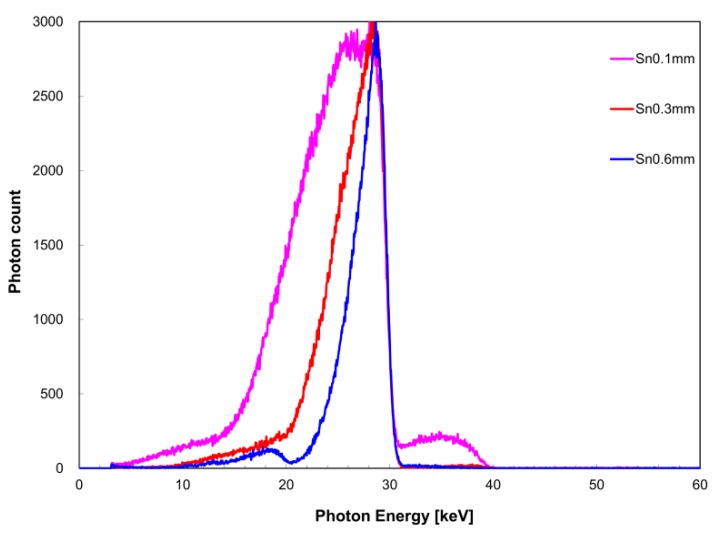
Filter thickness dependence of X-ray spectrum when the tin filter was used.

The measured X-ray spectrum with respect to filter thickness is shown in Figure 4 for the zirconium filter and Figure 5 for the tin filter. FWHM with respect to *I*(*E_K,Zr_*) was 4.1 keV for a filter thickness of 0.1 mm, 2.5 keV for 0.3 mm, and 1.9 keV for 0.6 mm. With respect to *I*(*E_K,Sn_*), FWHM was 9.4 keV for a filter thickness of 0.1 mm, 5.2 keV for 0.3 mm, and 3.2 keV for 0.6 mm. The measurement time was 259 s for the zirconium filter thickness of 0.1 mm, 267 s for 0.3 mm, and 1754 s for 0.6 mm. For the tin filter, the measurement time was 319 s for a filter thickness of 0.1 mm, 151 s for 0.3 mm, and 320 s for 0.6 mm.

### 3.3. Conditions to Achieve Quasi-Monochromatic Dual-Energy

**Figure 6:**
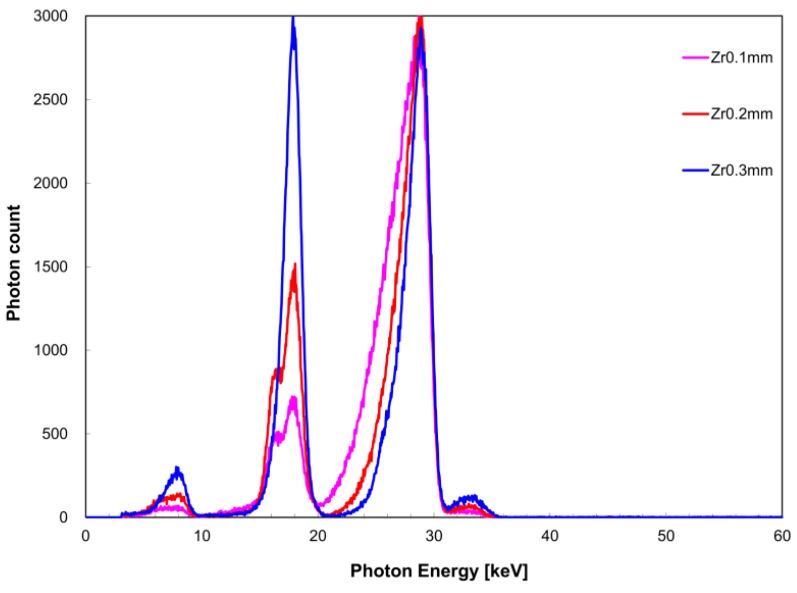
Zirconium filter thickness dependence of X-ray spectrum when the tin filter thickness was 0.2 mm.

**Figure 7:**
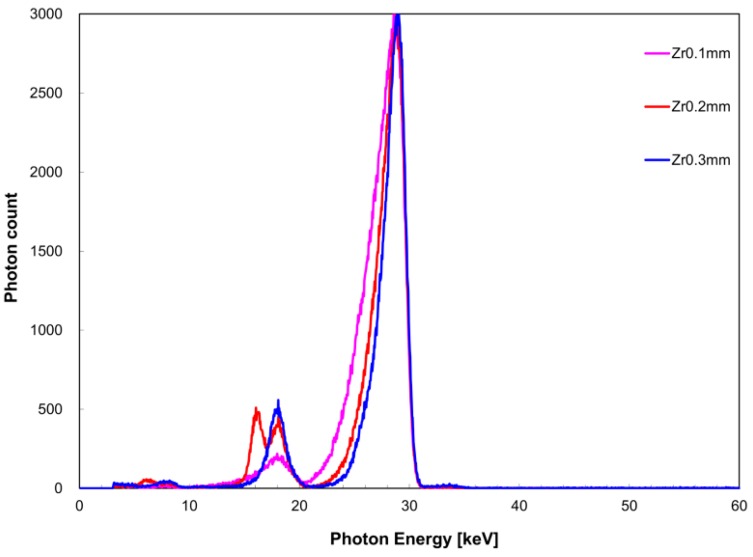
Zirconium filter thickness dependence of X-ray spectrum when the tin filter thickness was 0.3 mm.

Figure 6 shows the measured X-ray spectrum when the zirconium filter thickness was changed against a constant tin filter thickness of 0.2 mm. Similarly, Figure 7 shows the case where the tin filter thickness was 0.3 mm. When the tin filter thickness was 0.2 mm, FWHM with respect to *I*(*E_K,Zr_*) was 3.0 keV for a zirconium filter thickness of 0.1 mm, 2.6 keV for 0.2 mm, and 1.7 keV for 0.3 mm; and with respect to *I*(*E_K,Sn_*) was 3.9 keV for a zirconium filter thickness of 0.1 mm for, 2.9 keV for 0.2 mm, and 2.4 keV for 0.3 mm. When the tin filter thickness was 0.3 mm, FWHM with respect to *I*(*E_K,Zr_*) was 2.8 keV for a zirconium filter thickness of 0.1 mm, 3.4 keV for 0.2 mm, and 1.6 keV for 0.3 mm; and with respect to *I*(*E_K,Sn_*) was 3.9 keV for a zirconium filter thickness of 0.1 mm, 3.4 keV for 0.2 mm, and 2.2 keV for 0.3 mm. When the tin filter thickness was 0.2 mm, *I*(*E_K,Zr_*)/*I*(*E_K,Sn_*) was 0.24 for a 0.1-mm zirconium filter thickness, 0.51 for 0.2-mm thickness, and 1.0 for 0.3-mm thickness. Similarly, when the tin filter thickness was 0.3 mm, *I*(*E_K,Zr_*)/*I*(*E_K,Sn_*) was 0.073 for a 0.1-mm zirconium filter thickness, 0.15 for 0.2-mm thickness, and 0.19 for 0.3-mm thickness.

### 3.4. Measurement Accuracy of Mineral Density of Simulated Bone Tissue

**Figure 8:**
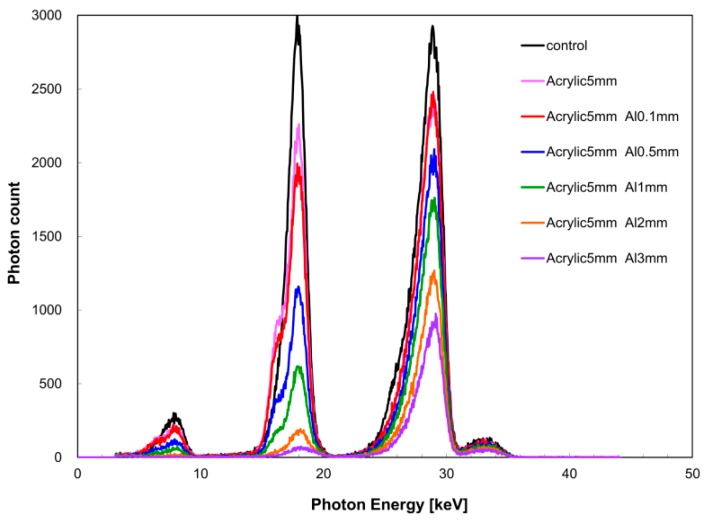
X-ray spectrum in aluminum density evaluation by dual-energy X-ray absorptiometry.

Figure 8 shows the change in the X-ray spectrum in terms of aluminum filter thickness, and Table 1 shows the measurement results of aluminum density. The relative error between aluminum density (measured value) obtained from the measured X-ray spectrum and the nominal value was within 5% at aluminum thicknesses of 1 and 2 mm.

## 4. Discussion

In the measured X-ray spectrum in terms of tube voltage, considering the FWHM value of *I*(*E_K_*), it appeared that the tube voltage was preferably higher than the K-absorbed edge energy because FWHM tended to decrease as the tube voltage increased. However, it was shown that when the maximum X-ray energy became greater than the K-absorption edge energy, the polyenergetic X-ray component (which cannot be attenuated by a filter) became noticeable. *I_K_*/*I* appeared to be less influenced by the polyenergetic X-ray components other than quasi-monochromatic, single-energy X-rays formed using the K-absorption edge filter as the value approached 1. These findings suggest that a tube voltage of 20 or 30 kV is appropriate for the zirconium filter, but 30 kV can be considered more appropriate in consideration of the FWHM data. In the case of the tin filter, *I_K_*/*I* was almost equal for tube voltages of 30 and 40 kV; therefore, 40 kV is considered equally suitable. Based on the above results, it appears that setting the tube voltage such that the maximum energy is higher by 10 keV than the K-absorption edge energy is necessary to obtain a quasi-monochromatic, single-energy X-ray.

In the measured X-ray spectrum with respect to filter thickness, it is desirable to have a large filter thickness because the FWHM value of *I*(*E_K_*) tended to inversely decrease with filter thickness increase. However, there is a concern that as the filter thickness increases, the measurement time is prolonged. Long measurement times should be avoided in consideration of the device load. Therefore, measurement time was considered while setting the conditions of filter thickness. Use of a zirconium filter thickness of 0.6 mm resulted in six-times longer measurement time than when a thickness of 0.1 or 0.3 mm was used. In fact, a tin filter thickness of 0.6 mm required more than twice the measurement time required for a tin filter thickness of 0.3 mm. Therefore, a filter thickness of 0.3 mm appears to be suitable.

From the above examinations, the optimal tube voltage for the acquisition of quasi-monochromatic, single-energy X-ray when a single filter was used was found to be 30 kV for the zirconium filter and 40 kV for the tin filter. Therefore, when a dual filter was used, an X-ray spectrum was acquired as an intermediate value at a tube voltage of 35 kV. For a tin filter thickness of 0.2 mm, we observed an improvement in monochromaticity of *I*(*E_K,Zr_*) and *I*(*E_K,Sn_*) with increasing thickness of the zirconium filter because the separation between the two energy peaks became more clear. In contrast, although the monochromaticity of *I*(*E_K,Sn_*) improved when the tin filter thickness was 0.3 mm compared to when it was 0.2 mm, it is considered inferior as dual peak X-ray spectrum because *I*(*E_K,Zr_*)/*I*(*E_K,Sn_*) had markedly reduced. Because the aim of this study was to perform a quantitative evaluation using the measured values of two energy peaks when a dual filter is used, the monochromaticities of each energy peak and X-ray intensity were kept uniform. Therefore, it appears that the combination of a zirconium filter thickness of 0.3 mm and tin filter thickness of 0.2 mm is optimal for the acquisition condition of the quasi-monochromatic, dual-energy X-ray with respect to a tube voltage of 35 kV.

In the quantitative evaluation of bone mineral density, aluminum density was calculated using the acquisition condition of quasi-monochromatic dual-energy X-ray as described above. In the aluminum thickness range of 1–3 mm, it was possible to perform calculations with a relative error within 5%, supporting its use as cited previously for measurement of knee cartilage thickness. When the aluminum thickness was less than 1 mm, deviation from the calculated value occurred because the measurement error became dominant with respect to the decrease in X-ray intensity. Therefore, based on the present study, if the aluminum thickness is less than 1 mm, it is necessary to conduct measurement in the low dual X-ray energy region. However, when the aluminum thickness is 3 mm or more, there is a concern of the energy peak of *I*(*E_K,Zr_*) not being recognized, thereby reducing its accuracy. The accuracy found is inferior in this study considering the clinical evaluation criteria [28], but previous studies have suggested an accuracy greater than that of MRI [29,30]. When performing quantitative evaluation of bone mineral density using DEXA, it is desirable to generate quasi-monochromatic, dual-energy X-rays in a suitable energy range in consideration of the X-ray absorption of the sample. In consideration of the effective atomic number, we used aluminum and polymethyl methacrylate resin as an alternative to bone and soft tissue, respectively, in our experiment. However, biomaterials have a different effective atomic number and exhibit complicated morphological structure. Measurement of bone mineral density using quasi-monochromatic, dual-energy X-ray is advantageous in that it is less susceptible to beam hardening compared with medical X-rays. However, we did not use regenerated bone tissue; this is a limitation of our study. Thus, the results of the measurement accuracy of bone mineral density obtained from a simulation experiment may not be adapted to various biomaterials. Although the results are in support of using DEXA using quasi-monochromatic, dual-energy X-ray to evaluate mineral density of regenerated bone tissue, its diagnostic value may be limited.

## 5. Conclusions

In the present study, we focused on the differences in terms of K-absorption edge energy and aimed to determine the optimal conditions of tube voltage and filter thickness to form a quasi-monochromatic energy spectrum using tin and zirconium filters. We found that it was most appropriate to set the tube voltage to achieve a maximum X-ray energy approximately 10 keV higher than the K-absorption edge energy to achieve quasi-monochromatic, single-energy X-rays. Additionally, using an intermediate value (35 kV) of the tube voltage to obtain the quasi-monochromatic single-energy X-ray for each filter, the optimal filter thickness was determined to be 0.3 mm for the tin filter and 0.2 mm for the zirconium filter. In bone mineral density measurement using the DEXA method with quasi-monochromatic, dual-energy X-rays, aluminum was used as a substitute for thin bone tissue, and we determined the measurement accuracy within a range of 1–3 mm with a relative error within 5%. The present study demonstrated that the quasi-monochromatic X-ray technique using the microfocus X-ray generator has higher versatility than conventional methods. However, it is necessary to consider X-ray energy depending on the target tissue, suggesting the possibility of obtaining important information for a bone mineral density evaluation indicator.

## Figures and Tables

**Table 1:** Aluminum density calculated from quasi-monochromatic, dual-energy X-ray spectrum.

Aluminum Thickness (mm)	Aluminum Density (g/cm^2^)		Relative Error (|[A]/[B]−1| × 100) (%)
	Actual Measurement (Mean Score of Measurement Performed Twice) [A]	Nominal Value [B]	
0.1	0.052	0.027	48.4
0.5	0.182	0.135	25.7
1	0.279	0.270	3.1
2	0.559	0.540	3.5
3	0.762	0.810	6.2
